# Reliability of Long Vein Grafts for Reconstruction of Massive Wounds

**DOI:** 10.3390/jcm12196209

**Published:** 2023-09-26

**Authors:** Brian Chuong, Kristopher Katira, Taylor Ramsay, John LoGiudice, Antony Martin

**Affiliations:** 1 College of Science Main Campus, University of Utah, Salt Lake City, UT 84112, USA; 2 Intermountain Healthcare, Salt Lake City, UT 84107, USA; 3 Salt Lake Community College, Main Campus, Salt Lake City, UT 84123, USA; 4 Department of Plastic and Reconstructive Surgery, Medical College of Wisconsin, Milwaukee, WI 53226, USA

**Keywords:** microsurgery, plastic surgery, free flap reconstruction, AV loops, end-to-side anastomoses

## Abstract

When handling large wounds, zone of injury is a key concept in reconstructive microsurgery, as it pertains to the selection of recipient vessels. Historically, surgeons have avoided placing microvascular anastomosis within widely traumatized, inflamed, or radiated fields. The harvest of vein grafts facilitates reconstruction in complex cases by extending arterial and/or venous pedicle length. To illustrate the utility and fidelity of these techniques, this paper reviews the indications and outcomes for vein grafting in ten consecutive patients at a single tertiary referral center hospital. The case series presented is unique in three aspects. First, there are two cases of successful coaptation of the flap artery to the side of the arterial limb of an arteriovenous loop. Second, there is a large proportion of cases where vein grafts were used to elongate the venous pedicle. In these 10 cases, the mean vein graft length was 37 cm. We observed zero flap failures and zero amputations. Although limited in sample size, these case data support the efficacy and reliability of long segment vein grafting in complex cases in referral centers.

## 1. Introduction

Microsurgery is the technical means through which free tissue transfer is accomplished. In large cancer and trauma referral centers, free tissue transfer involves using skin, muscle, and/or bone to restore massive soft tissue defects in the head and neck, trunk, and extremities. Naturally, executing a free flap requires meticulous planning, including careful flap and recipient vessel selection [1]. “Zone of injury” is a critical concept in this context, as heavily scarred, traumatized, or poorly perfused vessels do not nourish or drain a free flap, potentially leading to the dreaded complication of flap failure. In this regard, vein grafting is an important yet often under-recognized tool for successfully reconstructing even the most complex and heavily damaged wounds [2].

The following manuscript describes a case series of ten consecutive patients in a single tertiary referral center in which free tissue transfer was used to restore traumatic or oncologic defects. Descriptive details of the procedures, employed techniques, length of vein grafts used, and outcomes are provided as evidence of the efficacy, reliability, and reproducibility of large segment vein grafting in even the most intricate reconstructive cases. To bolster the significance of these findings, the outcomes from our case series are juxtaposed against the existing literature on long vein graft free flap tissue transfers [3,4,5,6,7]. In these referenced articles, patients underwent various forms of free tissue transfer involving vein grafting, with discussions on indications, complications, and success rates. This comparative analysis underscores the high levels of success in each of the cases presented, providing valuable insight and reinforcement into the utility of vein grafting as a vital tool in the arsenal of microsurgeons. 

## 2. Methods

Ten consecutive patient charts at a single institution (Intermountain Medical Center) over a two-year period were studied to elucidate details about the nature of the problems requiring reconstruction, the technical details of the operation, the size of the wounds, and the length of the vein grafts used. Complications such as hematoma, flap loss, microvascular thrombosis, re-operation, and time to soft tissue healing were recorded and tabulated. Institutional review board approval was obtained for the recording and publication of these data (ID: 1052396). All patient photos were de-identified to the fullest extent possible. Written consent for photograph release and for publication of these data were obtained. 

Vein grafts were used when flaps could not be inset using standard pedicle lengths, especially in cases involving heavily traumatized, scarred, or radiated fields. Consent was obtained from patients pre-operatively. Sonographic vein mapping was performed in one case using vascular lab services. Perioperative chemoprophylaxis was routinely administered. Non-constrictive and well-padded splinting was employed in cases involving extremities to avoid motion when long vein grafts spanned joint soft tissues. Vein grafts used were typically inset under wide subcutaneous tunnels to allow coaptation to recipient vessels. Arteriovenous (AV) loops were allowed flowing for at least 30 min before division, enabling the correction of vasospasm prior to flap coaptation. 

## 3. Results

**Figure 1:**
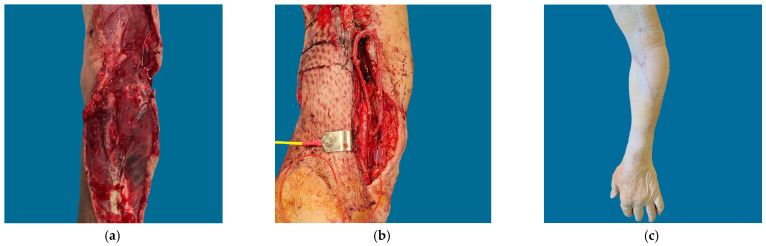
Patient B in Table 1 and Table 2. Free tissue transfer was performed for definitive wound coverage after extensive and prolonged degloving injury, allowing for future reconstructions of PIN palsy. A pedicled latissimus flap was used to cover a Gustilo Grade IIIa condylar fracture prior to attempting free tissue transfer, allowing for skin paddle placement over an antibiotic spacer. Dermal matrix was used to temporize the distal wound bed before free tissue transfer. (**a**) Defect; (**b**) Arteriovenous (AV) Loop creation; (**c**) 1 year follow-up.

**Figure 2:**
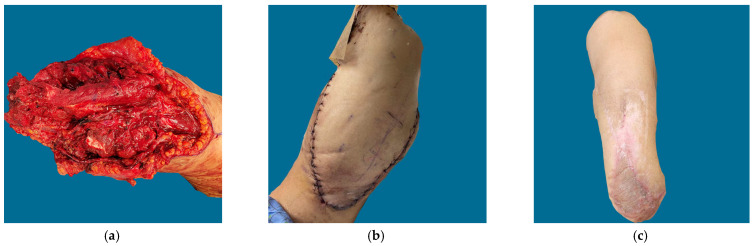
Patient D in Table 1 and Table 2. Industrial accident resulting in trans-radial amputation. A traditional AV loop was created to allow for elbow preservation and prosthetic fitting. (**a**) Defect with AV loop; (**b**) Immediate post op result after ALT flap; (**c**) Healed flap/skin graft at 6 months.

**Figure 3:**
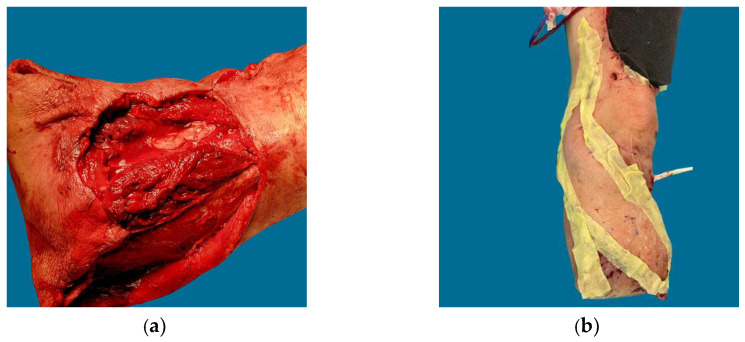

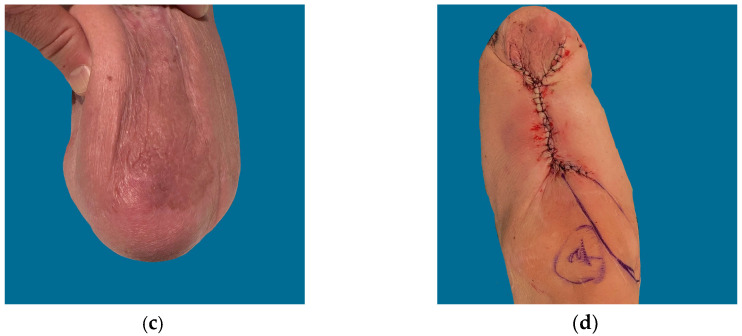
Patient A in Table 1 and Table 2. Pedestrian versus automobile accident resulting in traumatic amputation. (**a**) After internal fixation and antibiotic spacer; (**b**) Myocutaneous free tissue transfer was performed to preserve the below-knee amputation stump; (**c**,**d**) Flap elevation and advancement after Masquelet bone grafting.

**Figure 4:**
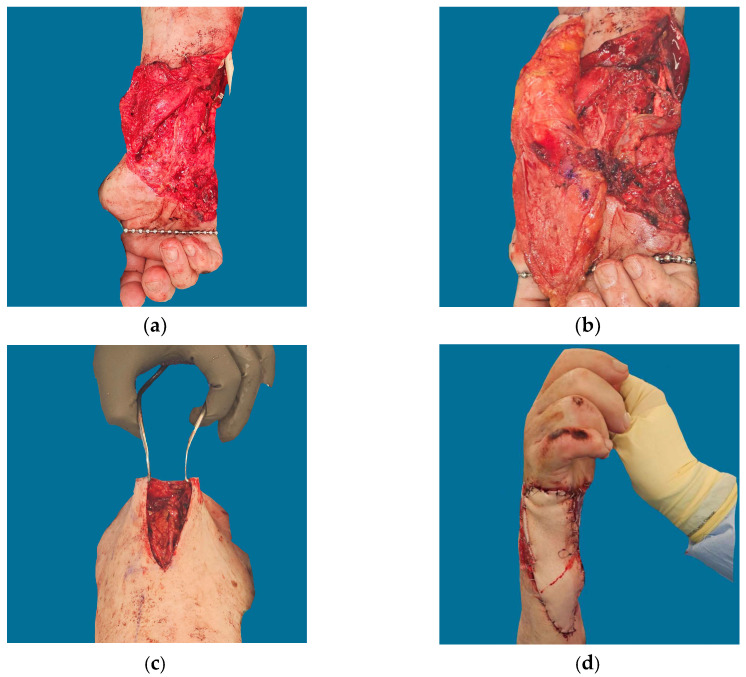
Patient F in Table 1 and Table 2. Extensive crush mechanism resulting in exposure of the ulnar nerve with flexor tendon injury. A long vein graft was used to bridge the flap vein to more proximal veins, providing outflow to large competent vessels outside of the zone of injury; (**a**) Defect; (**b**,**c**) Vein graft used to extend venous pedicle from flap to uninjured proximal antebrachial vein; (**d**) Final inset.

Figure 1, Figure 2, Figure 3 and Figure 4 depict cases in which long vein grafts were employed for various defects. The indications and techniques in this consecutive series of 10 patients are summarized in Table 1. The mean vein graft length was 37 cm. The most common vein graft donor site length was the thigh’s greater saphenous vein (9/10 cases). The most common flap used was the anterolateral thigh flap (ALT, N = 4), followed by latissimus (N = 2), vastus lateralis (N = 2), followed by gracilis (N = 1) and radial forearm (N = 1). Flap viability was 100% with a minimum follow-up of 3 months and a maximum follow-up of 18 months. Amputation was avoided in 100% of patients during this time. Nine cases were related to acute or subacute traumatic defects (within the same hospitalization as the index traumatic injury), and one case was related to radiation injury. One trauma-related case was several years removed from the injury. 

**Table 1:** Indications and Technique for Vein Graft Anastomosis.

Patient	Etiology	Indication	Location (Size, cm)	Flap	Vein Graft (cm)	Recipient Artery/Vein	Technique (Figure 5)
A	Pedestrian vs. MVC	IIIB Open Fracture	Anterior BKA Stump	MC Vastus Lateralis	GSV (25) Leg and Thigh	Medial Genicular a/GSV	Type 2
B	MVA Degloving	IIIA Open Fracture, PIN Palsy	Elbow Forearm, Arm	ALT	GSV (35)	Brachial a./Brachial V.	Type 3
C	MVA Degloving	IIIB Open Fracture	Circumferential Forearm	Latissimus	GSV (44)	Radial a./Brachial V.	Type 1
D	Industrial Machine	IIIC Fracture, Multiple Nerves Injured	Forearm	ALT	GSV (50)	Proximal Ulnar a./Brachial V.	Type 3
E	Sarcoma	Radiation	Tibia, Knee	Vastus Lateralis	GSV (70) Leg and Thigh	LCF a. and TFL Branch a./GSV	Type 4 for Artery Type 1 for Vein
F	Industrial Machine	Soft Tissue Degloving, Ulnar Nerve and Flexor Tendon Injuries	Wrist, Forearm	ALT	GSV (33)	Ulnar a./Brachial V.	ES a. Type 1 Vein
G	Infection	Exposed Tendon, Median Nerve	Wrist	ALT	LFC vc (25)	Radial a./Brachial V.	EE a. Type 1 Vein
H	Blast	Degloved Thumb	Hand	Gracilis	GSV (30)	Radial a./Radial V.C.	ES a. Type 1 Vein
I	Blast	Distal Third Extremity Wound, Remote Injury	Ankle	Radial Forearm	GSV (30)	Posterior Tibial a. Superficial Vein	EE a. Type 1 Vein

Index of Table 1: MVC—Motor Vehicle Collision. LFC—Lateral Femoral Circumflex. TFL—Tensor Fascia Lata. VC—Vena Comitans. ES—End to Side . EE—End to End. AV—Arteriovenous. PIN—Posterior Interosseous Nerve. BKA—Below the Knee Amputation. MC—Musculocutaneous. ALT—Anterolateral Thigh Flap. GSV—Greater Saphenous Vein.

Table 1 summarizes the indications, etiologies, and technical aspects of wound coverage in this series. Two surgical teams were used to execute these cases. A variety of vein graft techniques were employed, including three cases of traditional arteriovenous (AV) loop, two of which required end-to-side anastomosis of the flap vessel along the arterial limb of the vein graft. Two AV loop cases were connected to free flaps in a single stage, while the remaining case was performed in two stages to allow for scheduling of a definitive free flap. In the two-stage arteriovenous (AV) flap, the AV loops was left in place under skin flaps and then divided in a separate procedure. The two-stage technique in this case was applied for purely logistical reasons. 

Table 2 summarizes complications and outcomes related to these flap transfers. Unplanned takeback occurred in two patients due to venous anastomotic revision. In Patient A, two takebacks were required for drainage of flap site hematoma on Postoperative day (POD) 1 and venous revision on POD 2. Additionally, two procedures were performed for donor site wound closure and dermal regeneration template grafting in the same hospitalization. Patient E also required takeback for hematoma and revision of the venous anastomosis on POD 1. Patient C had one unplanned takeback following discharge to wash out a donor site seroma. Patient A also underwent skin grafting of a dermal regeneration template and two secondary orthopedic procedures after hospital discharge. There were no cases of major venous thromboembolism, flap loss, or revision amputation during the follow-up periods. The minimum follow-up was three months for one patient, while the rest of the patients had a follow-up duration of six months or more. 

**Table 2:** Complications and Outcome Vein Graft.

Patient	Takeback for Anastomosis Revision	Other Takeback	Thromboembolism (DVT/PE)	Soft Tissue Healed?	Post-Operative Amputation?	Follow-Up (Months)
A	Yes; Washout hematoma, thrombectomy, vein revision, washout and closure of flap donor site	Yes; Secondary Orthopedic Procedures	No	Yes	No	18
B	No	Yes; Skin Grafting of Wound, Excision of Neuroma	No	Yes	No	12
C	No	Yes; Donor Site Infection	No	Yes	No	12
D	No	No	No	Yes	No	6
E	Yes; washout hematoma, thrombectomy, vein revision	No	No	Yes	No	3
F	No	No	No	Yes	No	12
G	No	No	No	Yes	No	9
H	No	No	No	Yes	No	12
I	No	No	No	Yes	No	6

Multiple types of AV looping techniques exist. Type 1 construct consisted of a flap vein sewn or coupled end to end to a vein graft, and the downstream limb of the vein graft was then sewn or coupled end to end to the recipient vein. There was a total of seven Type 1 vein grafts in seven patients. All seven cases were for efferent connections.

Type 2 and Type 3 constructs comprised the arteriovenous loop vein graft group. Type 2 involved creation of a traditional AV loop, in which an end-to-end connection was formed between the vein graft and the recipient artery and vein. This loop was then divided in the same surgery (one-stage AV loop) or in a separate procedure (two-stage), creating afferent and efferent vein segments which were then connected end to end to flap vessels. Type 3 involved a size-mismatched AV loop. In this type, a standard AV loop was created as in Type 2. Unlike Type 2, however, the flap artery was sewn end to side, rather than end to end, to the afferent vein graft stump at the time of loop division. Two AV loop cases (one Type 2, one Type 3) were connected to free flaps in a single stage, while the remaining case (Type 3) was performed in two stages to allow scheduling of a definitive free flap. In the two-stage AV loop, the arteriovenous fistula was left in place under skin flaps and then divided in a separate procedure six days later to allow for OR free flap scheduling.

Type 4 consisted of a Y shaped connection to recipient vessels, using vein graft branch points to supercharge flow to or from the flap. Type 4 vein graft case referenced in Table 1 (Patient E) involved arterial supercharging by connecting both the descending lateral femoral circumflex (DLFC) and the tensor fascia lata (TFL) pedicle arteries to branches of a Y shaped saphenous graft to cover a knee wound.

**Figure 5:**
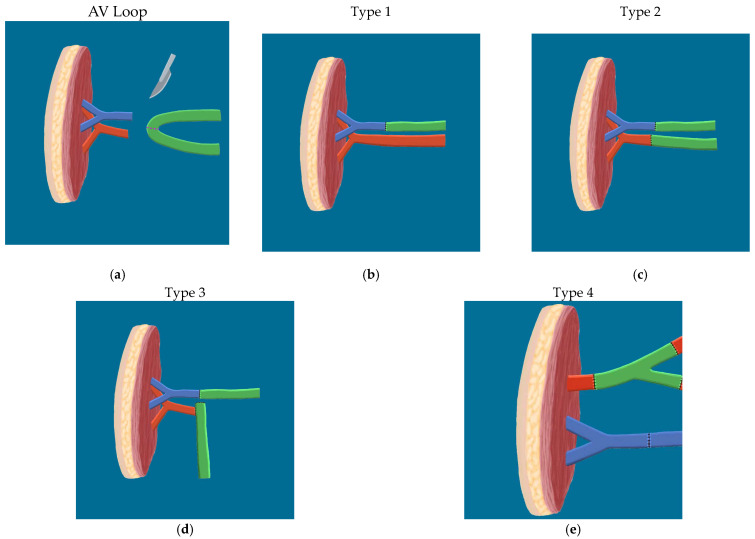
Illustrations showcasing various forms of anastomosis. (**a**) Initial AV loop creation, purple dotted line represents splitting of loop; (**b**) Type 1 end-to-end venous anastomosis; (**c**) Type 2, traditional AV loop anastomosis into vein and artery; (**d**) Type 3, mismatched AV loop with end-to-side arterial anastomosis with a stump. End-to-end venous anastomosis; (**e**) Type 4, arterial branch anastomosis allowing for increased arterial blood flow.

The diagrams in Figure 5 summarize the techniques used for anastomosis, expanding upon various vein graft patterns used for extending pedicle length to reliable vessels for free tissue transfer. 

## 4. Discussion

This case series demonstrates the feasibility of massive flap reconstructions in heavily traumatized wounds using vein grafts as well as the reliability of these techniques across multiple surgeons. Three different surgeons at the same institution were involved in the care of these patients, and excellent outcomes were achieved in terms of flap viability, limb salvage, and amputation-free postoperative courses. Despite the increase in operative time required for vein graft harvest and vascular anastomosis, long segment vein grafting led to successful outcomes in this particular series. All patients exhibited dependable soft tissue healing and experienced successful follow-up, resulting in a 100% salvage rate and a 0% need for post-flap revision amputation. These outcomes compare favorably to the success rates reported in the established literature. Notably, Bost et al., Henn et al., Momeni et al., and Brumberg et al. all reported complex cases involving interpositional vein grafts and arteriovenous (AV) loops, with flap failure rates ranging from 3% to 20.3% [3,4,5,6,7]. Moreover, Lin et al., and Brumberg et al., who also explored limb salvage outcomes, reported impressive amputation-free survival rates of 83–93% and 90%, respectively [5,6,7]. In this practice, vein grafting was used in approximately 10% of free flap cases. In general, longer operative times were required for vein loop harvest cases compared to other free flap cases, but major venous thromboembolism was not encountered in any patients. Vein graft harvest itself adds approximately one to two hours of increased operative time for graft harvest and closure, as well as the need for additional microvascular anastomosis. 

Takebacks or unplanned operations occurred in both acute and delayed post-operative phases. In the immediate post-operative period, venous thrombosis presented in the form of an expanding hematoma, which serves as a sentinel sign of venous compromise [8]. The existing literature underscores the importance of restoration and management of perfusion to ensure successful outcomes [9]. In both cases, mechanical causes were identified as reasons for the venous clot. The rate of re-exploration due to anastomotic causes was 20% in this small sample, a figure consistent with findings in prior literature. For instance, in Bos et al.’s series involving 90 interposition vein grafts and AV loops across 56 patients, 10 of the 42 vein-grafted flaps necessitated takeback for emergent salvage, with successful salvage achieved in 7 of these cases. In the same study, five two-stage vein grafts thrombosed before free flap transfer occurred [3]. Henn et al. presented a series of 103 cases involving arteriovenous loops, with observed thrombosis rates of 11–14%, major complication rates of 26–30%, and flap failure rates of 7–11% [4]. Lin et al. reported a case series encompassing 65 arteriovenous loops and interposition grafts for arterial pedicle elongation, revealing re-exploration rates ranging from 22% to 43% [5]. Additionally, Momeni et al. detailed one instance of re-exploration for arterial thrombosis in an AV loop case [6]. In this series, as in the case series referenced above, early re-exploration allowed for thrombectomy, excision of any damaged segment of vein graft, and clearance of any residual thrombus using tissue plasminogen activator drugs [10]. Additional experience, fluid resuscitation, and judicious use of chemoprophylaxis agents perioperatively could lead to a reduction in the incidence of these outcomes in future patients. One late, unplanned operation was performed for donor site seroma, a known complication of latissimus flap harvest. As additional cases were performed in the case series, the rate of unplanned takebacks decreased.

The outcomes and procedures in this case series are comparable to those in the established literature for vein or AV loop grafts for free tissue transfer. Bos et al. and Hen et al. presented a series of single- and two-stage AV loops with flap failure rates of 6%, and vein graft thrombectomy rates were reported to be higher for delayed two-stage AV loop cases [3,4]. Lin et al. presented a series of 65 cases of vein grafts longer than 20 cm for reconstruction and note re-exploration rates of 20–30% in select subgroups of cases [5]. In Momeni et al.’s series of 20 cases, outcomes for 10 AV-Loop free flaps were compared against 10 matched free flaps without vein grafts. Single-stage AV loop creation was found to have similar outcomes as those of free flaps that did not require vein grafting [6]. Brumberg et al. presented a series of 10 AV loop vein grafts for mangled lower extremity cases and noted amputation for infection in one case and 100% loop patency. In their series, AV loops were reported to be long, as corroborated by the practice of anastomosing loops to the popliteal and superficial femoral vessels for leg coverage [7]. The absence of thromboembolic events in our case series can be attributed to perioperative chemoprophylaxis use in this practice, but these data are not widely discussed in previous vein graft/free flap papers. While this practice is not universally implemented in reconstructive microsurgery cases, there is an increasing awareness of the importance of this clinical practice throughout plastic surgery [11].

In addition to reporting the reliability of these techniques, this case series demonstrates the versatility of vein grafts and highlights the feasibility of an end to side anastomosis to a stump on a vein graft. Four different vein graft constructs were implemented in this case series (Figure 5), and the use of vein grafts for venous pedicle extension was prevalent. While extending vein grafts to inflow pedicles remains a viable option, the collective experience of this group with extensive degloving and radiation injuries suggests that the quality of outflow vessels was comparatively less optimal (posing challenges in dissection due to fragility and small caliber) when compared to inflow vessels within the zone of injury. Vein grafting allowed for outflow to larger caliber veins in multiple instances. The use of vein grafts for venous pedicle extension alone is not widely represented in the literature, as most papers focus on arterial pedicle extension or AV loop creation.

Perhaps a common but under-reported practice among surgeons, arterial or venous supercharging can be accomplished using vein graft branch points (Figure 5, Type 4) [12]. In the one Type 4 case in this series, vasospasm was encountered along the DLFC system and TFL supercharging reliably improved flap perfusion and served as an adjunct to traditional vasospasm relieving techniques, such as use of topical calcium channel blockers, adventitial stripping, and warm heparinized saline irrigation. Similar practices are employed for augmenting venous outflow in the breast reconstruction along with head and neck literature to improve venous outflow [13,14]. 

In Figure 5, Type 3 AV loop patterns are noteworthy as a viable microsurgical salvage technique since neither case required anastomotic revision. The indication for this technique is a size mismatch between graft and flap arteries, a common phenomenon in saphenous loops connected to high inflow systems. In one case, a venotomy was required to directly sew the flap artery to the vein graft; a side branch of a vein graft was required in the other. The surgeons in this group reserved the Type 3 technique for inflow only, as a mismatch between thin, pliable veins may be more easily overcome compared to flap artery and vein graft size mismatches using traditional end-to-end anastomotic techniques. Venous vein graft segments demonstrate slower flow physiology than arterial vein graft limbs, which may increase the risk of venous thrombosis if there is turbulent flow within a stump adjacent to a venous anastomosis. In any case, an end-to-side arterial anastomosis to a stump of vein graft is a technique reported in the cardiac literature in cases of bypass grafting and should be employed if needed to complete the reconstruction. End-to-side and side-to-side anastomoses were demonstrated and juxtaposed as methods for linking small target coronary arteries to vein bypass grafts, with comparative analysis [15]. 

As acknowledged by multiple authors in the reconstructive literature, the thigh is an excellent reconstructive tissue bank [16]. The thigh donor site stood out as the predominant location utilized for flap harvesting. In general, dependable perforators are typically situated along the descending axis. In cases where the associated morbidity is considered acceptable, flaps derived from the tensor fasciae latae (TFL), vastus, or rectus muscles can serve as viable options [17]. When the wound size is relatively small in comparison to the extent of injury in the distal lower extremity, opting for the radial forearm flap can obviate the need for vein graft harvesting. Although a considerable amount of literature discusses the morbidity associated with the radial forearm flap, strategies for mitigating its impact have also been explored. Non-dominant forearm site harvest, use of an adipofascial flap design, placement of dermal regeneration templates over donor site flexor tendons, reconstruction of the radial artery and pre-operative and intraoperative pulse-oximetry Allen’s testing have all led to acceptable outcomes in select patients [18]. Wounds with a large surface area of exposed critical structure can be covered with the latissimus flap, obviating the need for a vein graft [19]. The relatively larger surface area of this flap effectively lengthens the flap pedicle in addition to providing coverage of larger wounds. Nevertheless, in two instances within this series of latissimus flap procedures, vein graft augmentation was employed to extend the pedicle, underscoring the substantial magnitude of the degloving injuries observed in this particular case collection. 

Even though saphenous veins were preferred as a source of grafts in this case series (90% of patients), deep vein or arterial grafts can be used when superficial veins are unusable or unavailable. The thigh tissue bank is also interesting because it can provide superficial, deep arterial, or venous grafts to elongate flap pedicle length [20]. As shown in case H, the venae comitantes of the descending lateral circumflex system can be used instead of saphenous grafts. In this manner, a second donor site can be avoided and reconstruction can be offered to patients with sclerosed or traumatized superficial veins, such as in cases of infection, degloving, or following venipuncture procedures. We found that the lumina of superficial veins are of adequate caliber to match to those of workhorse flap venae and are particularly useful when grafting is required for preserving outflow. That said, saphenous vein grafts are still the predominant source of vein grafts in the literature [21]. 

Several shortcomings of this data set should be acknowledged. The small sample size allows for the possibility that the reliability of vein grafting is not as high as reported in this case series of ten patients. However, the technical adaptability and remarkable success rates achieved within this cohort of patients showcases the efficacy of these methodologies, which, in terms of outcomes and intricacy, are on par with the literature on vein graft free flap procedures. Lin et al. reported an average vein graft length extension of 26–32 cm for elongating arterial pedicles, whereas in this study, the mean extension reached 37 cm [5]. Meanwhile, Brumberg et al. made reference to the use of long vein grafts in arteriovenous loops originating “at or above the knee” without specifying vein graft lengths [7]. This study also sheds light on the employment of vein grafts to supercharge arterial pedicles via branch points and successfully demonstrates end-to-side anastomosis with long segment vein grafts, both of which represent innovative arrangements that expand upon the established techniques of AV loop creation, which typically involves the creation of an arteriovenous fistula with end-to-end coaptations between the flap vessels and an interpositional vein graft [5,7]. Cavadas et al. presented a case involving bifurcated greater saphenous vein for double venous flow coaptation following AV loop creation, akin to the arterial supercharging seen in this series’ Type 4 construct [22]. Moreover, the notably high proportion of vein grafts employed for extending venous pedicles (N = 5 in 10 cases) within this case series is a distinctive feature, as previous authors have predominantly described their use for either AV loop creation or arterial pedicle elongation [3,4,5,6,7]. While acknowledging the limitation of the small sample size in this case series, the 100% flap success rate and the absence of amputations among the associated patients, akin to the limb salvage rates in Lin et al. and Brumberg et al.’s vein graft series, provides compelling support for the utilization of these innovative techniques within this patient population. From that standpoint, this case series is valuable in the microsurgical vein graft literature. Another shortcoming of these data set is that operative time was not measured and could not be compared to matched cases without designing prospective databases on these cases. Besides expanding the number of patients in this series, future studies could focus on the morbidity of vein graft harvest, quantify wound measurements, compare operative time for vein graft harvest compared to matched cases, and detail long-term functionality of salvage patients using standardized protocols in this patient population [23,24].

## Data Availability

The data presented in this study are available on request from the corresponding author. The data are not publicly available due to patient privacy.
